# Supplementary material for: Synaptic connectome of a neurosecretory network in the Drosophila brain
Source: eLife. 2026 Oct 12;13:RP102684. doi: 10.7554/eLife.102684 (PMC13657632; doi:10.7554/eLife.102684)
Supplement: Supplementary file 2 [file elife-102684-supp1.docx]

**Supplementary File 1:** Fly strains used in this study.

| **Fly strain** | **BDSC Stock number / Reference** |
| --- | --- |
| *DH44-Gal4* | 51987 |
| *DILP2-Gal4* | (Ikeya et al. 2002) |
| *DILP3-Gal4* |  |
| *DILP5-Gal4* | 66008 |
| *MS-T2A-Gal4* | 84652 |
| *Gr64a-Gal4* | 57661 |
| *CRZ-Gal4* | 51976 |
| *R24F06-p65.AD/CyO; R45E06-DBD/TM6B (DNg27-split-Gal4)* | 75837 |
| *empty-split-Gal4* | 79603 |
| *empty-Gal4* | 68384 |
| *w^1118^* | 5905 |
| *UAS-retro-Tango* | (Sorkac et al. 2023) |
| *JFRC81-10xUAS-IVS-Syn21-GFP-p10 (EGFP)* | (Pfeiffer, Truman, and Rubin 2012) |
| *10xUAS-myr::GFP (myrGFP)* | (Pfeiffer et al. 2010) |
| *UAS-NLS-mCherry* | 38425 |
| *20xUAS-CsChrimson::mVenus* | Janelia Farms |
| *UAS-Kir2.1::EGFP* | 6595 |

Ikeya, T., M. Galic, P. Belawat, K. Nairz, and E. Hafen. 2002. 'Nutrient-dependent expression of insulin-like peptides from neuroendocrine cells in the CNS contributes to growth regulation in *Drosophila*', *Curr Biol*, 12: 1293-300.

Pfeiffer, B. D., T. T. Ngo, K. L. Hibbard, C. Murphy, A. Jenett, J. W. Truman, and G. M. Rubin. 2010. 'Refinement of tools for targeted gene expression in *Drosophila*', *Genetics*, 186: 735-55.

Pfeiffer, B. D., J. W. Truman, and G. M. Rubin. 2012. 'Using translational enhancers to increase transgene expression in *Drosophila*', *Proc Natl Acad Sci U S A*, 109: 6626-31.

Sorkac, A., R. A. Mosneanu, A. M. Crown, D. Savas, A. M. Okoro, E. Memis, M. Talay, and G. Barnea. 2023. 'retro-Tango enables versatile retrograde circuit tracing in *Drosophila*', *Elife*, 12.
