# Supplementary material for: Synaptic connectome of a neurosecretory network in the Drosophila brain
Source: eLife. 2026 Oct 12;13:RP102684. doi: 10.7554/eLife.102684 (PMC13657632; doi:10.7554/eLife.102684)
Supplement: Supplementary file 3 [file elife-102684-supp2.docx]

**Supplementary File 2:** Antibodies used for immunohistochemistry in this study.

| **Antibody** | **Immunogen** | **Dilution** | **Source / Reference** |
| --- | --- | --- | --- |
| ***Primary antibodies*** | | | |
| chicken anti-GFP | Recombinant full-length GFP | 1:1000 | Abcam, RRID: AB_300798 |
| chicken anti-GFP | Recombinant full-length GFP | 1:1000 | Rockland (#600-901-215) |
| guinea pig anti-RFP |  | 1:5000 | Gift from Dr. Susan Morton |
| mouse nc82 anti-Bruchpilot |  | 1:50 | (Wagh et al. 2006) |
| rat anti-HA (3F10) | YPYDVPDYA | 1:100 | Roche |
| rabbit anti-CRZ | *Periplaneta americana* CRZ | 1:1000 | (Veenstra and Davis 1993) |
| rabbit anti-DILP2 | *Drosophila* DILP2 | 1:2000 | (Veenstra, Agricola, and Sellami 2008) |
| rabbit anti-DILP3 | *Drosophila* DILP3 | 1:500 | (Veenstra, Agricola, and Sellami 2008) |
| rabbit anti-MS | *Locusta migratoria* MS | 1:500 | (Schoofs et al. 1993) |
| rabbit anti-CAPA | *Rhodnius prolixus* CAPA-2 | 1:2000 | Gift from Dr. Ian Orchard |
| ***Secondary antibodies*** | | | |
| goat anti-chicken Alexa Fluor® 488 | IgY (H+L) chicken | 1:1000 | Thermo Fisher Scientific |
| donkey anti-guinea pig Alexa Fluor® 555 |  | 1:1000 | Thermo Fisher Scientific |
| goat anti-mouse Alexa Fluor® 647+ |  | 1:1000 | Thermo Fisher Scientific |
| goat anti-guinea pig Alexa Fluor® 647 |  | 1:1000 | Thermo Fisher Scientific |
| donkey anti-rabbit Alexa Fluor® 555 |  | 1:1000 | Thermo Fisher Scientific |
| donkey anti-rat Alexa Fluor® 555 |  | 1:1000 | Thermo Fisher Scientific |
| donkey anti-rabbit Alexa Fluor® 647 |  | 1:1000 | Thermo Fisher Scientific |
| donkey anti-mouse Alexa Fluor® 647 |  | 1:1000 | Thermo Fisher Scientific |

Schoofs, L., G. M. Holman, L. Paemen, D. Veelaert, M. Amelinckx, and A. De Loof. 1993. 'Isolation, identification, and synthesis of PDVDHFLRFamide (SchistoFLRFamide) in Locusta migratoria and its association with the male accessory glands, the salivary glands, the heart, and the oviduct', *Peptides*, 14: 409-21.

Veenstra, J. A., H. J. Agricola, and A. Sellami. 2008. 'Regulatory peptides in fruit fly midgut', *Cell Tissue Res*, 334: 499-516.

Veenstra, J. A., and N. T. Davis. 1993. 'Localization of corazonin in the nervous system of the cockroach *Periplaneta americana*', *Cell Tissue Res*, 274: 57-64.

Wagh, D. A., T. M. Rasse, E. Asan, A. Hofbauer, I. Schwenkert, H. Durrbeck, S. Buchner, M. C. Dabauvalle, M. Schmidt, G. Qin, C. Wichmann, R. Kittel, S. J. Sigrist, and E. Buchner. 2006. 'Bruchpilot, a protein with homology to ELKS/CAST, is required for structural integrity and function of synaptic active zones in *Drosophila*', *Neuron*, 49: 833-44.
