# Supplementary material for: Synaptic connectome of a neurosecretory network in the Drosophila brain
Source: eLife. 2026 Oct 12;13:RP102684. doi: 10.7554/eLife.102684 (PMC13657632; doi:10.7554/eLife.102684)
Supplement: Peer review history

# Editor report: eLife Assessment

**DOI:** 10.7554/eLife.102684.3.sa0

**Authors:** John C Tuthill

**Affiliations:** University of Washington United States

**Keywords:** Convincing, Important

This study uses **convincing** analyses of publicly available connectomic and transcriptomic datasets to survey the anatomy and connectivity of neurosecretory cells in the *Drosophila* brain. The systems-level description of the neurosecretory system is complemented by selected functional experiments and predictions for circuit function and paracrine signaling networks that can now be tested in future studies. This **important** work will be of interest to neuroscientists working on hormonal signaling in *Drosophila* and other animals.

# Referee report: Reviewer #1 (Public review)

**DOI:** 10.7554/eLife.102684.3.sa1

Summary:

The study by McKim et al (eLife-RP-RA-2024-102684) seeks to provide a comprehensive description of the connectivity of neurosecretory cells (NSCs) using a high-resolution electron microscopy dataset of the fly brain and several single cell RNA seq transcriptomic datasets from the brain and peripheral tissues of the fly. They use connectomic analyses to identify discrete functional subgroups of NSCs and describe both the broad architecture of the synaptic inputs to these subgroups as well as some of the specific inputs including from chemosensory pathways. They then demonstrate that NSCs have very few traditional presynapses consistent with their known function as providing paracrine release of neuropeptides. Acknowledging that EM datasets can't account for paracrine release, the authors use several scRNAseq datasets to explore signaling between NSCs and characterize widespread patterns of neuropeptide receptor expression across the brain and several body tissues. The thoroughness of this study allows it to largely achieve its goal and provides a useful resource for anyone studying neurohormonal signaling.

Strengths:

The strengths of this study are the thorough nature of the approach and the integration of several large-scale datasets to address shortcomings of individual datasets. The study also acknowledges the limitations that inherent to studying hormonal signaling and provide interpretations within the context of these limitations.

# Referee report: Reviewer #2 (Public review)

**DOI:** 10.7554/eLife.102684.3.sa2

Summary:

The authors provide a comprehensive description of the neurosecretory network in the adult *Drosophila* brain. They assigned and verified the types of neurosecretory cells (NSCs) found in three publicly available *Drosophila* brain connectomes. They then describe the organization of synaptic inputs and outputs for across NSC types. They show that NSCs are regulated by multiple sensory modalities, including enteric neurons. The authors then focus on a concise pathway from corazonin-expressing NSCs to a set of descending neurons, DNg27 and demonstrate that this pathway has the capacity to regulate egg-laying in female flies. Leveraging existing transcriptomic data, they also describe the hormone and receptor expressions in the NSCs and show putative paracrine signaling between NSCs. Taken together, this study provides a framework for future functional experiments, which may demonstrate whether and how NSCs, and the circuits to which they belong, shape physiological function and behavior.

Strengths:

This study uses three *Drosophila* brain connectomes to assign cell types to ten classes of neurosecretory cells (NSCs), based on clustering of synaptic connectivity and morphological features. The authors then verify type assignments for selected populations by matching cluster sizes to anatomical localization and cell counts using immunohistochemistry of neuropeptide expression and markers with known co-expression.

The authors compare their findings to previous work describing the synaptic connectivity of the neurosecretory network in larval *Drosophila* (Huckesfeld et al., 2021), finding that there are some differences between these developmental stages. Direct comparisons between adult and larvae are made possible through direct comparison in Table 1, as well as the authors' choice to adopt similar (or equivalent) analyses and data visualizations in the present paper's figures.

The authors extract core themes in NSC synaptic connectivity and generate predictions regarding sensory inputs and downstream physiological and behavioral functions. They test one newly identified NSC-premotor pathway, from corazonin-expressing NSCs to the descending neuron DNg27, with loss-of-function experiments and demonstrate that this pathway has the capacity to regulate female egg-laying.

The authors illustrate expression patterns of neuropeptides and receptors across NSC cell types from existing transcriptomic data and present a putative paracrine signaling network among NSCs. The authors also catalog hormone receptor expression across tissues.

Taken together, this study provides a comprehensive account of the neurosecretory system of the adult fly.

Weaknesses:

In Figure 6 authors use a linear dynamical modeling approach (described in Bates et al. 2026) to quantify the influence of different sensory source neuron types on the different NSC classes. The authors should discuss the two main assumptions baked into this approach: (1) all path segments (connections) from sources to targets are given the same sign and therefore result in activation, despite likely biological variation in their synaptic valences. (2) Each connection is given the same time constant for the response kinetics. Therefore, the model assumes uniform intrinsic "biophysical" properties.

Although the actual intrinsic properties (e.g. complements of voltage-gated ion channels) of the intermediate and target neurons are unknown, they are likely heterogenous. Such heterogeneity would have consequences on the steady-state responses. Thus, the response magnitudes measured in this model are unlikely to provide an accurate representation of feedforward "influences" in this circuit.

Although the intrinsic properties of all nodes in these paths will remain unknown in the absence of electrophysiological recordings, one could still consider the signs of connections using neurotransmitter predictions in the connectome (Eckstein et al. 2024). It would then be useful to compare the relative influences calculated with the Bates et al. approach to (1) simple weight propagation methods which are agnostic to time (as in Hoeller et al. 2026; doi: https://doi.org/10.64898/2025.12.22.696097) and (2) this Bates et al. approach and weight propagation methods that conserve the signs of the connections.

In Figure 8 and associated supplements, the authors probe the function of CRZ-expressing NSCs > DNg27 pathways in female and male flies. Although the authors test the effects of silencing both CRZ-expressing cells and DNg27 on feeding, egg-laying, and flight behaviors in females. They recapitulate a previous finding that CRZ-expressing cells regulate feeding behavior and then identify potential regulatory roles for this pathway in egg-laying. However, the authors did not test this full palette of behaviors in males. The authors do not test feeding or flight behaviors in males. They do, however, confirm previously reported activation phenotypes (copulation-like behaviors), via optogenetic activation of CRZ-expressing cells in males. These experiments would be more ethological if executed in freely walking male flies, rather than males that were glued, on their backs. It is unclear why the authors did not also test for activation or loss-of-function phenotypes for DNg27 in males. Taken together: the authors show compelling loss-of-function phenotypes for feeding and egg-laying for the CRZ-expressing NSC > DNg27 pathway in females, but evaluation in males remains incomplete.

# Author comment: Author response

**DOI:** 10.7554/eLife.102684.3.sa3

**Authors:** Theresa H McKim, Jayati Gera, Ariana J Gayban, Kevin Christie, Joel Shin, Nils Reinhard, Giulia Manoli, Selina Hilpert, Patrick Callaerts, Floris van Breugel, Charlotte Helfrich-Förster, Meet Zandawala

**Affiliations:** University of Nevada, Reno Reno United States; University of Würzburg Würzburg Germany; University of Nevada, Reno Reno United States; University of Nevada, Reno Reno United States; University of Nevada, Reno Reno United States; University of Würzburg Würzburg Germany; University of Würzburg Würzburg Germany; University of Würzburg Würzburg Germany; KU Leuven Leuven Belgium; University of Nevada, Reno Reno United States; University of Wuerzburg Wuerzburg Germany; University of Nevada, Reno Reno United States

The following is the authors’ response to the original reviews.

**eLife Assessment**

This valuable study uses the analysis of connectomic and transcriptomic datasets to survey the anatomy and connectivity of neurosecretory cells in the *Drosophila* brain. While the connectivity analyses are convincing, the anatomical and functional data provided to verify cell type identity and paracrine signaling is incomplete. Once these aspects are improved, this study would be of interest to neuroscientists working on hormonal signaling in *Drosophila* and other animals.

We thank the editor and reviewers for their assessment of our manuscript. We hope that the additional results in the revised manuscript addresses all of the concerns.

**Public Reviews:**

**Reviewer #1 (Public review):**

Summary:

The study by McKim et al seeks to provide a comprehensive description of the connectivity of neurosecretory cells (NSCs) using a high-resolution electron microscopy dataset of the fly brain and several single-cell RNA seq transcriptomic datasets from the brain and peripheral tissues of the fly. They use connectomic analyses to identify discrete functional subgroups of NSCs and describe both the broad architecture of the synaptic inputs to these subgroups as well as some of the specific inputs including from chemosensory pathways. They then demonstrate that NSCs have very few traditional presynapses consistent with their known function as providing paracrine release of neuropeptides. Acknowledging that EM datasets can't account for paracrine release, the authors use several scRNAseq datasets to explore signaling between NSCs and characterize widespread patterns of neuropeptide receptor expression across the brain and several body tissues. The thoroughness of this study allows it to largely achieve it's goal and provides a useful resource for anyone studying neurohormonal signaling.

Strengths:

The strengths of this study are the thorough nature of the approach and the integration of several large-scale datasets to address short-comings of individual datasets. The study also acknowledges the limitations that are inherent to studying hormonal signaling and provides interpretations within the context of these limitations.

We thank this reviewer for the thorough assessment and highlighting the strengths of our manuscript. Based on comments from the other reviewer, we now include additional analyses of NSCs from two new recent datasets – the brain and nerve cord (BANC) connectome and the male central nervous system (maleCNS) connectome. Our original conclusions based on the FlyWire connectome remain unchanged, further validating our analyses.

Weaknesses:

Overall, the framing of this paper needs to be shifted from statements of what was done to what was found. Each subsection, and the narrative within each, is framed on topics such as "synaptic output pathways from NSC" when there are clear and impactful findings such as "NSCs have sparse synaptic output". Framing the manuscript in this way allows the reader to identify broad takeaways that are applicable to other model system. Otherwise, the manuscript risks being encyclopedic in nature. An overall synthesis of the results would help provide the larger context within which this study falls.

We agree with the reviewer and have modified the subsection titles to highlight the main findings within those sections.

We have also included a figure (new Figure 10) which summarizes the main findings from our manuscript and places them within the larger context of neuroendocrine signaling in adult *Drosophila* in relation to other studies.

The cartoon schematic in Figure 5A (which is adapted from a 2020 review) has an error. This schematic depicts uniglomerular projection neurons of the antennal lobe projecting directly to the lateral horn (without synapsing in the mushroom bodies) and multiglomerular projection neurons projecting to the mushroom bodies and then lateral horn. This should be reversed (uniglomerular PNs synapse in the calyx and then further project to the LH and multiglomerular PNs project along the mlACT directly to the LH) and is nicely depicted in a Strutz et al 2014 publication in eLife.

We thank the reviewer for spotting this error. We have now modified the schematic as suggested.

**Reviewer #2 (Public review):**

Summary:

The authors aim to provide a comprehensive description of the neurosecretory network in the adult *Drosophila* brain. They sought to assign and verify the types of 80 neurosecretory cells (NSCs) found in the publicly available FlyWire female brain connectome. They then describe the organization of synaptic inputs and outputs across NSC types and outline circuits by which olfaction may regulate NSCs, and by which Corazon-producing NSCs may regulate flight behavior. Leveraging existing transcriptomic data, they also describe the hormone and receptor expressions in the NSCs and suggest putative paracrine signaling between NSCs. Taken together, these analyses provide a framework for future experiments, which may demonstrate whether and how NSCs, and the circuits to which they belong, may shape physiological function or animal behavior.

Strengths:

This study uses the FlyWire female brain connectome (Dorkenwald et al. 2023) to assign putative cell types to the 80 neurosecretory cells (NSCs) based on clustering of synaptic connectivity and morphological features. The authors then verify type assignments for selected populations by matching cluster sizes to anatomical localization and cell counts using immunohistochemistry of neuropeptide expression and markers with known co-expression.

The authors compare their findings to previous work describing the synaptic connectivity of the neurosecretory network in larval *Drosophila* (Huckesfeld et al., 2021), finding that there are some differences between these developmental stages. Direct comparisons between adults and larvae are made possible through direct comparison in Table 1, as well as the authors' choice to adopt similar (or equivalent) analyses and data visualizations in the present paper's figures.

The authors extract core themes in NSC synaptic connectivity that speak to their function: different NSC types are downstream of shared presynaptic outputs, suggesting the possibility of joint or coordinated activation, depending on upstream activity. NSCs receive some but not all modalities of sensory input. NSCs have more synaptic inputs than outputs, suggesting they predominantly influence neuronal and whole-body physiology through paracrine and endocrine signaling.

The authors outline synaptic pathways by which olfactory inputs may influence NSC activity and by which Corazonin-releasing NSCs may regulate flight. These analyses provide a basis for future experiments, which may demonstrate whether and how such circuits shape physiological function or animal behavior.

The authors extract expression patterns of neuropeptides and receptors across NSC cell types from existing transcriptomic data (Davie et al., 2018) and present the hypothesis that NSCs could be interconnected via paracrine signaling. The authors also catalog hormone receptor expression across tissues, drawing from the Fly Cell Atlas (Li et al., 2022).

We thank this reviewer for the thorough assessment and for highlighting the strengths of our manuscript. Based on comments from the other reviewer, we now include additional analyses of NSCs from two new recent datasets – the brain and nerve cord (BANC) connectome and the male central nervous system (maleCNS) connectome. Our original conclusions based on the FlyWire connectome remain unchanged, further validating our analyses.

Weaknesses:

The clustering of NSCs by their presynaptic inputs and morphological features, along with corroboration with their anatomical locations, distinguished some, but not all cell types. The authors attempt to distinguish cell types using additional methodologies: immunohistochemistry (Figure 2), retrograde trans-synaptic labeling, and characterization of dense core vesicle characteristics in the FlyWire dataset (Figure 1, Supplement 1). However, these corroborating experiments often lacked experimental replicates, were not rigorously quantified, and/or were presented as singular images from individual animals or even individual cells of interest. The assignments of DH44 and DMS types remain particularly unconvincing.

We thank the reviewer for this comment. We would like to clarify that all immunohistochemical images presented in this manuscript are representative images based on at least 5 independent samples. We have now clarified this in the methods.

Additionally, we show *DH44* > *retro-Tango* signal across five samples (new Figure 2 Supplement 3) to highlight the consistency of retrograde trans-synaptic labeling. We also show the neurons providing inputs to putative m-NSC^DH44^ and putative m-NSC^DMS^ in both FAFB and maleCNS connectomes (new Figure 2 Supplement 2B-C). In both the FAFB and maleCNS datasets, we see a group of neurons (marked by black arrows) providing inputs to m-NSC^DMS^ but not m-NSC^DH44^. Importantly, these input neurons are not labelled in *DH44* > *retro-Tango* samples, lending further support to our assignment of DH44 and DMS cell types.

The electron micrographs showing dense core vesicle (DCV) characteristics (new Figure 2 Supplement 2E-G) are also representative images based on examination of multiple neurons. However, we agree with the reviewer that a rigorous quantification would be useful to showcase the differences between DCVs from NSC subtypes. Therefore, we have now performed a quantitative analysis of the DCVs in putative m-NSC^DH44^ (n=6), putative m-NSC^DMS^ (n=6) and descending neurons (n=2) known to express DMS across three datasets (FlyWire, BANC and maleCNS connectomes). For consistency, we examined the cross section of each cell where the diameter of nuclei was the largest. We quantified the mean gray value of at least 50 DCVs per cell. The individual who performed these analyses was blind to the neuron identity. Our analysis (new Figure 2 Supplement 2H-J) shows that mean gray values of putative m-NSC^DMS^ and DMS descending neurons in FAFB and maleCNS are not significantly different, whereas the mean gray values of m-NSC^DH44^ are significantly higher. This analysis agrees with our initial DH44 and DMS NSC subtype assignments. Nonetheless, given the similarity in morphology and synaptic connectivity of DH44 and DMS neurons, we have included the limitation on cell type assignment in the absence of molecular markers in the connectome datasets.

The authors present connectivity diagrams for visualization of putative paracrine signaling between NSCs based on their peptide and receptor expression patterns. These transcriptomic data alone are inadequate for drawing these conclusions, and these connectivity diagrams are untested hypotheses rather than results. The authors do discuss this in the Discussion section.

We agree with the reviewer that the novel paracrine pathways presented are untested hypotheses. However, there is a very high likelihood that a given NSC subtype can signal to another NSC subtype using a neuropeptide if its receptor is expressed in the target NSC. This is due to the fact that all NSC axons are part of the same nerve bundle (nervi corpora cardiaca) which exits the brain. The axons of different NSCs form release sites that are extremely close to each other. While the release sites in NSCs cannot be visualized in adult *Drosophila* connectomes (since these regions were not included in the sample prep), these have been mapped in the larvae and shown to be in close proximity (Hückesfeld et al., 2021: https://doi.org/10.7554/eLife.65745). Neuropeptides from these release sites can easily diffuse via the hemolymph to peripheral tissues (e.g. fat body and ovaries) that are much further away from the release sites on neighboring NSCs. We believe that neuropeptide receptors are expressed in NSCs near these release sites where they can receive inputs, not just from the adjacent NSCs, but also from other sources such as the gut enteroendocrine cells. Hence, neuropeptide diffusion is not a limiting factor preventing paracrine signaling between NSCs, and receptor expression is a good indicator for putative paracrine signaling. Consistent with this, several pathways highlighted in the plot (CRZ to CAPA, DH44 to Hugin and Hugin to DH44) have been anatomically and/or functionally validated previously (Zandawala et al., 2021: https://doi.org/10.1371/journal.pgen.1009425; King et al., 2017: https://doi.org/10.1016/j.cub.2017.05.089; Mizuno et al., 2021: https://doi.org/10.1111/dgd.12733). Additionally, a similar analysis was also employed to depict putative interactions between NSCs in larval *Drosophila* (Hückesfeld et al., 2021). We have now modified the caption for this figure to explicitly state these connections are putative. We hope that the putative pathways presented here will inspire future functional studies, and have also highlighted this outstanding question in the summary Figure 10.

**Reviewer #3 (Public review):**

Summary:

The manuscript presents an ambitious and comprehensive synaptic connectome of neurosecretory cells (NSC) in the *Drosophila* brain, which highlights the neural circuits underlying hormonal regulation of physiology and behaviour. The authors use EM-based connectomics, retrograde tracing, and previously characterised single-cell transcriptomic data. The goal was to map the inputs to and outputs from NSCs, revealing novel interactions between sensory, motor, and neurosecretory systems. The results are of great value for the field of neuroendocrinology, with implications for understanding how hormonal signals integrate with brain function to coordinate physiology.

The manuscript is well-written and provides novel insights into the neurosecretory connectome in the adult *Drosophila* brain. Some, additional behavioural experiments will significantly strengthen the conclusions.

Strengths:

(1) Rigorous anatomical analysis

(2) Novel insights on the wiring logic of the neurosecretory cells.

We thank this reviewer for the thorough assessment and highlighting the strengths of our manuscript.

Weaknesses:

(1) Functional validation of findings would greatly improve the manuscript.

We agree with this reviewer that assessing the functional output from NSCs would improve the manuscript. Given that we currently lack genetic tools to measure hormone levels and that behaviors and physiology are modulated by NSCs on slow timescales, it is difficult to assess the immediate functional impact of the sensory inputs to NSC using approaches such as optogenetics. However, since l-NSC^CRZ^ are the only known cell type that provide output to descending neurons, we have functionally tested this output pathway using different behavioral assays (new Figure 8 and Supplements). Our analysis identifies a novel role for l-NSC^CRZ^ and DNg27 neurons in female reproduction (based on the number of eggs laid).

**Recommendations for the authors:**

**Reviewing Editor Comments:**

You will see that the reviewers found your work interesting and valuable, but had some suggestions for how revision could improve the manuscript. A common thread in the reviews is that functional speculations about the extracted circuits and paracrine signaling would benefit from revision, and would fit better in the Discussion, not Results section. Caveats could be more explicitly stated and language asserting functionality could be tempered. The reviewers were unanimous in their desire for a summary diagram or model.

We thank the editor for these suggestions to improve the manuscript. We have now functionally validated some output pathways from l-NSC^CRZ^. We have also toned down the language regarding functionality where appropriate. Finally, we included a figure (new Figure 10) which summarizes the main findings from our manuscript and places them within the larger context of neuroendocrine signaling in adult *Drosophila* in relation to other studies.

**Reviewer #2 (Recommendations for the authors):**

Suggestions for improved or additional experiments, data, or analyses:

The authors present connectomic analyses for NSCs identified in the FlyWire dataset. All of their connectomic findings would be strengthened by executing these same analyses in the freely available female hemibrain connectome (Scheffer et al. 2020; Plaza et al. 2022), thereby effectively increasing their sample size from one whole brain to three hemispheres. It is unclear why the authors chose only to focus on the FlyWire dataset.

We thank the reviewer for this suggestion. We had performed a preliminary analysis using the hemibrain dataset. However, out of the 80 endocrine cells that we found in FlyWire, the hemibrain dataset lacks both the NSC subtypes in the SEZ (SEZ-NSC^CAPA^ and SEZ-NSC^Hugin^) as well as l-NSC subtypes in the other hemisphere (l-NSC^ITP^, l-NSC^DH31^, l-NSC^CRZ^). In addition, a majority of the input synapses for all NSC are in the SEZ region which allowed us to classify the different NSC subtypes in FlyWire. Since this information is missing in the hemibrain dataset, we are unable to classify the m-NSC into the different subtypes (not shown). Therefore, we cannot perform a comprehensive analysis of input and output pathways of different NSC subtypes using the hemibrain dataset. To address this concern, we have repeated several analyses with two new recent datasets – the brain and nerve cord (BANC) connectome and the male central nervous system (maleCNS) connectome (Table 1, new Figure 1 Supplement 1, new Figure 2 Supplement 2, new Figure 3 Supplement 3, new Figure 6 Supplement 1, new Figure 7 Supplement 3). Our original conclusions based on the FlyWire connectome remain unchanged, further validating our analyses.

The authors initially map assign NSC types based on anatomical locations and clustering of presynaptic connections and morphological features. Due to matching cell counts and similar soma locations, they find that DMS and DH44 types cannot be easily distinguished. The authors attempt to assign cell types to these two populations using two methods, neither of which are convincing as executed:

(1) The authors attempt to distinguish the identities of the two populations by anatomically comparing presynaptic inputs in FlyWire to those observed with light microscopy using retrograde trans-synaptic labeling. Due to the lack of a genetic driver line for the DMS population, the authors could complete this only for the DH44 population. The authors present only one animal, at inadequate magnification to see the absence of distinguishing presynaptic neurons. The results would be strengthened by the presentation and quantification of multiple samples; without more than one sample, it is not possible to know how robust this finding is in this genetic driver line. The authors might also consider taking advantage of the widely-used template brain (Bogovic, 2020) to align their light micrographs of presynaptic inputs from the retrograde tracing, with the presynaptic skeletons from FlyWire and compare in a more quantitative and precise manner. The authors might also consider taking a similar approach using anterograde tracing (Talay et al. 2017) to label postsynaptic outputs. Given that postsynaptic outputs are fewer, so long as there are identifiable, distinct postsynaptic partners, it may be easier to distinguish the two populations with anterograde tracing.

We thank the reviewer for this comment. We would like to clarify that all immunohistochemical images presented in this manuscript are representative images based on at least 5 independent samples. We have now clarified this in the methods.

Additionally, we show *DH44* > *retro-Tango* signal across five samples (new Figure 2 Supplement 3) to highlight the consistency of retrograde trans-synaptic labeling. We also provide a magnified image in this figure to highlight the absence of presynaptic neurons that distinguish m-NSC^DH44^ and m-NSC^DMS^.

We also show the neurons providing inputs to putative m-NSC^DH44^ and putative m-NSC^DMS^ in both FAFB and maleCNS connectomes (new Figure 2 Supplement 2B-C). In both the FAFB and maleCNS datasets, we see a group of neurons (marked by black arrows) providing inputs to mNSC^DMS^ but not m-NSC^DH44^. Importantly, these input neurons are not labelled in *DH44* > *retroTango* samples, lending further support to our assignment of DH44 and DMS cell types.

As per this reviewer’s suggestion, we also aligned our retrograde tracing light micrographs to a template brain (Author response image 1). However, we were unable to quantitatively compare neurons in our light micrographs with neuronal skeletons from the connectome. This is because retroTango labels several neurons in the SEZ which obscures morphology of individual neurons needed for such comparisons. Additional experiments, where retro-Tango output is restricted to sparse populations of neurons using a Flp-out strategy, are needed to perform such quantitative analyses. These experiments are beyond the scope of this study since we now provide additional lines of evidence for cell assignments.

**Author response image 1.** DH44 > retro-Tango presynaptic signal aligned to JRC2018 unisex template brain.

We appreciate the suggestion to use the anterograde tracing tool trans-Tango to distinguish mNSC^DH44^ and m-NSC^DMS^. There is very little synaptic output from m-NSC^DMS^ and m-NSC^DH44^ based on the FlyWire connectome. There is no synaptic output from both of these cell types if we use a threshold of 5 synapses for significant connections (new Figure 7). Using a threshold of 2 synapses for significant synaptic connections, 3 neurons are downstream of m-NSC^DH441^ and 5 neurons are downstream of m-NSC^DMS^ (not shown). Since these postsynaptic neurons are not bilaterally paired (we do not anticipate unilateral pathways), we don’t think that these connections are significant. Consistent with our analysis with the FlyWire connectome, we did not observe any significant post-synaptic signal with DH44 > trans-Tango (Author response image 2) even using flies raised at 21ºC which increases the synaptic strength during development. Since we do not have a GAL4 driver to specifically target m-NSC^DMS^ , we could not perform similar trans-Tango analysis of m-NSC^DMS^.

**Author response image 2.** DH44 > trans-Tango (left) and w^1118^ > trans-Tango (right; control). Presynaptic neurons are labelled in green and post-synaptic neurons are in red. Representative images based on 5 samples.

Our connectome analyses revealed that putative m-NSC^DMS^ receive direct synaptic inputs from enteric neurons but m-NSC^DH44^ do not. We used this information to perform another trans-Tango analysis using Gr43a-Gal4 which labels a subpopulation of enteric neurons (Miyamoto and Amrein, 2013: https://doi.org/10.4161/fly.27241) (Author response image 3).

**Author response image 3.** Initiating trans-Tango from Gr43a neurons (green) does not label any postsynaptic neurons (magenta) in the pars intercerebralis (white arrow head), including those labelled by the DMS antibody (cyan).

Unfortunately, initiating trans-Tango from Gr43a neurons did not label any post-synaptic neurons in the pars intercerebralis where m-NSC^DH44^ and m-NSC^DMS^ soma are located. This could be due to (a) low trans-Tango sensitivity or (b) m-NSC^DMS^ are downstream from other enteric neurons not captured by Gr43a-GAL4. In the absence of other broad enteric neuron drivers, we are unable to perform additional analyses.

(2) The authors attempt to assign cell types by qualitatively assessing the darkness of dense core vesicles in these two populations. However, there is a presentation of only single planar images through three selected cells (a DMS-expressing descending neuron, DMS-expressing NSC, and DH44-expressing NSC) without any quantitative analyses of vesicle characteristics within or across NSC cell types. It is not possible for the reader to assess whether the darker vesicles constitute a real trend, or if these images are hand-selected to support their point. This piece of evidence would be more convincing if the authors demonstrate consistent vesicle characteristics within NSC type and differences across type. Moreover, such analysis of dense core vesicle features in cell types with distinct and known peptide expression would be broadly interesting.

Given that NSC type assignment is a major contribution of the present paper, it is critical that the authors are clear about the remaining uncertainty in assigning cell types, so as not to propagate false certainty into future work.

This comment has been addressed above, and we refer the reviewer to the new Figure 2 Supplement 2E-J.

The authors suggest larger peptide release capacity from CAPA-producing NSCs based on their larger morphological features (Figure 1, Supplement 2), which is more speculative than certain. In Figure 1, Supplement 1 the authors demonstrate the capacity to visualize vesicles number and size in individual NSCs. Rather than speculate over larger peptide release capacity based on cell size, the authors could quantify these vesicle features, which are surely a better indication of peptide release capacities.

We thank the reviewer for this comment. We agree that number of dense core vesicles within these and other neurons would be a better indicator of their peptide release capacity. We are performing these analyses on a brain-wide scale as part of another project. Therefore, we have removed the following speculative statement from the present manuscript:

“But given their location, large size, and presumed large release capacity, we speculate that SEZ-NSC^CAPA^ participate in global modulation of post-feeding physiology.”

The authors provide an analysis of NSCs' synaptic inputs and outputs, but never mention whether NSCs are synaptically connected to each other. If connected, it would be very sensible to provide some analysis of synaptic connectivity between NSCs. If they are not connected, the authors should explicitly mention this in the main text, as it is relevant to the overall aim of this study.

All NSCs are classified as endocrine cells in the FlyWire connectome. Hence, as shown in new Figures 3B and 7B, NSCs do not provide output to any endocrine cells (NSCs) using a threshold of 5 synapses for significant connections. Similarly, we do not see any synaptic connectivity between NSCs in the BANC dataset (new Figure 7 Supplement 3A-B). We do observe sparse connectivity between NSCs in the maleCNS dataset with a threshold of 5 synapses (new Figure 7 Supplement 3C-D), as well as in the Flywire connectome when the threshold is reduced to 2 synapses (new Figure 7 Supplement 2). However, we refrain from emphasizing on these connections because additional validation is required to rule out false positives in synapse predictions. Dense-core vesicles in NSCs can frequently be mistaken for synaptic T-bars during the prediction (unpublished observation).

Although unlikely, NSCs could also form synapses with each other near their release sites and outside the brain volumes captured in all three datasets examined in this study. This limitation has been included in the discussion.

There is no substitute for a good circuit wiring diagram; the motifs that are extracted in Figure 3H might be better appreciated if the reader was first presented with a well-formatted complete circuit diagram, which may then foreshadow the points made in the main text and in Figure 3H.

We appreciate this suggestion. We now include a circuit diagram (new Figure 3G) to highlight the connectivity between NSCs and their presynaptic partners. The proportion plot (old Figure 3G) has now been moved to new Figure 3 Supplement 5.

The authors provide extensive bar graphs showing synaptic input body IDs in Figure 3 Supplement 2, however they don't complete the same analysis for synaptic outputs (likely due to low numbers). Even so, it would be useful to the reader if the body IDs and cell types for both synaptic inputs and outputs were documented in a supplemental table. Providing such an inventory is aligned with the goals of this study.

Only l-NSC^unknown^ and l-NSC^CRZ^ provide synaptic outputs in the FlyWire connectome (new Figure 7B-F). We have included bar graphs showing output from both these cell types at a single-cell level (new Figure 7G). Additionally, we have annotated all the NSC subtypes in FlyWire and BANC on Codex. Further exploring the inputs and outputs of NSC subtypes can be done interactively on Codex. For example, the search command “{upstream_union} cell_type == SEZ_NSC_CAPA” will retrieve all the neurons providing inputs to SEZ-NSC^CAPA^. As a quick search, this is more convenient than pasting individual body IDs from a supplementary table into Codex. All code outputs (csv files) containing this information are also available on Zenodo.

In describing possible paracrine signaling, the authors write "Given the proximity of NSC axon terminations, it is extremely likely that a hormone released from a given NSC will influence the activity of other NSC types if its receptor is expressed in those cells." In the absence of functional experiments and/or information about spatial localization and/or peptide diffusion and the proximity of receptors to release sites, the expression patterns alone are insufficient to support this conclusion. Thus, the authors might consider removing the circular connectivity plots in Figure 7C, and Figure 7, Supplement 1A-H, and instead emphasize what can be concluded with certainty from the transcriptomic data (which are expression patterns of the hormones and receptors across NSCs and other tissue types). The authors might instead speculate over potential paracrine signaling between NSCs in the Discussion. Given that the authors describe paracrine signaling between NSCs as 'putative' in the abstract and main text, the authors will likely agree the legend for Figure 7 is misleading.

This comment has been addressed above. We agree with the reviewer and have modified the figure legends (new Figure 9 and Figure 9 Supplement 1) to emphasize that the connections are putative.

Should the authors keep these connectivity diagrams, it is important to reconsider their threshold wherein 50% of cells in a cluster must express a given hormone for it to be considered present in their analysis. It is entirely conceivable that there is real heterogeneity in hormone expression within the cluster, so it is surprising the authors have applied this artificial criterion.

We thank the reviewer for presenting us with this option.

We also apologize for the oversight in explaining our thresholding carefully. To minimize false positives, neuropeptides were subjected to a two-step filtering process. First, only those expressed in at least 50% of the cells within a given cluster were retained. Second, a composite expression score was calculated for each neuropeptide by multiplying its average expression by its percent detection. These values were normalized to the maximum observed signal across the dataset, and only hormone-cluster pairs maintaining a relative score of 0.25 or higher were included in the final analysis. This stringent filtering approach was implemented to focus the analysis on dominant neuropeptides and to exclude contamination from ambient RNA, which is common for neuropeptides (Allen et al., 2020: https://doi.org/10.7554/eLife.54074). To account for lower abundance of receptor transcripts, we used a more permissive threshold for receptors by retaining those expressed in at least 5% of the cells within a cluster. Unlike the neuropeptides, no secondary relative-score filtering was applied to the receptors to ensure that biologically relevant signaling targets were not prematurely excluded due to low transcript density. We have now revised our methods to explain these details.

Importantly, we used this thresholding criteria to align previous anatomical studies with our single-cell expression analysis and filter out neuropeptides that likely represent contamination: (1) Transcript for leucokinin (Lk) neuropeptide is expressed in l-NSC^ITP^ but previous studies have not been able to detect this peptide in l-NSC^ITP^ (Zandawala et al., 2018: https://doi.org/10.1371/journal.pgen.1007767). (2) Hugin is not expressed in m-NSC^DMS^ (Oh et al., 2021: https://doi.org/10.1016/j.neuron.2021.04.028). (3) Ilp2 is not expressed in SEZNSC^CAPA^ and m-NSC^DMS^ (this study and various others). (4) ITP is not expressed in any m-NSC (Gera et al., 2025: https://doi.org/10.7554/eLife.97043.3). Based on these and other examples, we feel that our stringent criteria recover putative pathways that are likely functional while filtering obvious false positive. Nonetheless, we agree with the reviewer that some of these NSCs could represent heterogeneous clusters as has been shown recently for m-NSC^DILP^ (Held, Bisen, Zandawala et al., 2025: https://doi.org/10.7554/eLife.99548.3). This heterogeneity could result in some authentic connections to drop out. However, our goal for this analysis was to not identify all the putative paracrine connections, but rather the strongest ones with the hope that it can inspire future functional studies.

The data shown in Figure 2 would be easier to interpret and therefore more convincing with better use of insets, appropriate overlays of multiple markers, higher image magnifications, and quantification across samples. Specifically: In Figure 2C, authors show mCherry expression but it isn't clear where these cell bodies are located with respect to the image in Figure 2B. This is also true for Figure 2D. Insets in Figure 2B that correspond with regions shown in 2C and 2D would be helpful. In Figure 2, the authors do not provide cell counts across samples for all markers. Thus, it isn't clear how consistent these cell counts are across samples. In Figure 2E, authors claim that no Gr64f-positive cells innervate the NCC, yet there is clearly a GFP signal in the NCC region in the merged image. The authors should provide an additional marker or a higher magnification image to convince the reader that these projections are not in the NCC region.

We thank the reviewer for these suggestions. To improve clarity, we have made the following changes:

Figures 2C and 2D are based on different samples than the one shown in Figure 2B. But we have added dashed boxes in Figure 2B to indicate the regions shown in Figure 2C and 2D.

Included sample sizes in Figure 2A and 2C. The rest of the panels are representative images based on at least 5 samples. This has been included in the methods.

The cell count has been provided for m-NSC^DILP^ for both markers in Figure 2A. The cell counts for m-NSC^DMS^, labelled using mCherry alone, has been provided in Figure 2C. We did not perform cell counting when using the membrane GFP reporter as it is difficult to accurately count overlapping cells (see dashed box labelled C in Figure 2B).

We also provide a new supplementary file (new Figure 2 Supplement 1) showing cell counts for m-NSC^DILP^ using different markers. Based on this, we can confidently conclude that adult *Drosophila* typically have more than 14 m-NSC^DILP^.

We have corrected a typo in our label for Figure 2E: it should be Gr64a instead of Gr64f.

We have modified the Figure 2E inset to show that the four pairs of Gr64a > myrGFP expressing corazonin cells do not project via the NCC (labelled with an arrow). We have also identified the four pairs of Gr64a neurons (Author response image 4 left panel) in the FlyWire connectome, which shows that these neurons do not exit the brain via the NCC.

**Author response image 4.** Corazonin-expressing Gr64a neurons in the FlyWire connectome (left) and a light micrograph (right, same as in Figure 2E) showing Gr64a neurons (green) and corazonin neurons (magenta).

Recommendations for improving the writing and presentation:

Throughout the paper, the authors provide scant or, at times, no citations. Inadequate citation is as much an issue in the introduction as it is in the results and discussion sections. As such, the authors often do not provide a well-supported premise for the present work and/or do not place their findings and interpretations into the context of existing literature. Related, there is a predominance of references to the work of the authors themselves, often in place of citing earlier foundational work. Citations are nearly exclusive to the *Drosophila* literature, with the exception of the second paragraph of the introduction. This paper would be greatly improved with references to a broader literature.

We have now added additional references to give credit to foundational work where appropriate. We have also included citations to non-*Drosophila* literature for more general statements in the introduction and discussion; however, we refrain from citing such studies in the results section to keep it focused.

Figure 1 Supplement 1 is referenced after Figure 2 in the text. The authors might consider reassigning it as a supplement to Figure 2, which also uses imaging methodologies to distinguish NSC cell types.

We agree with the reviewer and have reassigned the figures accordingly.

Figure 3G is difficult to interpret, and its figure legend is brief and inadequate.

As suggested by this reviewer, we have replaced this panel with a circuit diagram. The proportion plot (old Figure 3G) has now been moved to Figure 3 Supplement 5, and we have expanded the figure legend.

The bar graphs in Figure 3 Supplements 2 and 3 would best benefit the reader if the x-axis labels are not simply body IDs, but also cell types or instances (if assigned in FlyWire).

We appreciate this suggestion. Cell types are routinely updated on Codex while the root IDs remain static for v783. Therefore, we chose root IDs for these plots as they can be used to query Codex easily and reliably. We now provide all raw data as csv files on Zenodo used to make these plots. This includes cell types and other classifications.

Minor corrections to the text and figures:

Table 1 compares the observed numbers of NSC types in adult flies to those in larvae and those expected based on previous literature. The authors should cite the previous studies that support each of the expected or larval numbers, either within the table or in the table legend. It would also be appreciated if the expected numbers were cited in the main text.

References for NSC numbers in larvae and expected numbers in adults are now included in Table 1.

In describing the author's approach to analyzing synaptic connectivity by cosine similarity, authors cite their own previous work rather than the foundational study describing this approach or earlier studies that use it.

We have now also cited Schlegel et al., 2021 (https://doi.org/10.7554/eLife.66018) who used a similar approach in the olfactory system.

**Reviewer #3 (Recommendations for the authors):**

(1) The observation that most gustatory inputs to NSCs are indirect (particularly for feedingrelated NSCs) is very interesting but lacks functional validation. I suggest that the authors conduct behavioural assays where specific sensory inputs are activated or silenced while monitoring outputs from NSCs. This could include optogenetics to stimulate or inhibit sensory neurons, or alternative feeding assays.

We thank the reviewer for this insightful suggestion. We agree that the functional validation of gustatory-to-NSC pathways is a highly compelling direction for future research. However, we believe that behavioral assays, as suggested, pose significant interpretive challenges for the following reasons:

NSCs primarily function by releasing hormones into the systemic circulation. Unlike classical neurotransmission, hormonal modulation typically operates on much slower timescales (minutes to hours). Consequently, acute activation of sensory inputs is unlikely to elicit immediate, quantifiable behavioral changes that can be specifically attributed to NSC activity.

Most NSC classes are known to influence multiple physiological and behavioral processes simultaneously. Attributing a specific behavioral phenotype to a single NSC class following sensory stimulation would be confounded by these overlapping roles.

Activating or silencing taste neurons will directly impact feeding behavior through canonical motor circuits, independent of the neuroendocrine system. In such a paradigm, it would be nearly impossible to isolate the specific "indirect" contribution of the NSCs to the observed behavior.

While we agree that functional connectivity, such as optogenetic activation of taste neurons paired with calcium imaging (e.g., GCaMP) in NSCs, would be the ideal way to validate these inputs, we consider these extensive physiological experiments to be beyond the scope of this anatomical and connectomic study.

Nonetheless, to address this important question, we now use a recently developed approach (Bates et al., 2026: https://doi.org/10.1101/2025.07.31.667571) based on linear dynamical modeling to estimate the influence of various sensory neurons (gustatory, olfactory, enteric, hygrosensory, etc.) on different NSC classes. Our analysis (new Figure 6 and Figure 6 Supplement 1) reveals that contents of consumed food (detected by enteric neurons) have a stronger influence on NSCs compared to inputs from external taste receptors.

(2) Descending neurons appear to play a crucial role in regulating both motor and endocrine output. However, their functional contribution is only inferred from the connectomic data. The authors could perform functional activity manipulations (silencing or activating) of these descending neurons (for instance dMS descending neurons) to explore their role in behaviour. This could be tested with simple behavioural assays such as feeding or reproduction (i.e egg laying).

We believe that there might be some confusion. DMS descending neurons (DNp32 cell type) used for dense-core vesicle comparisons with m-NSC^DMS^ and m-NSC^DH44^ (new Figure 2 Supplement 2) are different from the descending neurons (DNg27 cell type) that receive inputs from l-NSC^CRZ^ (new Figure 7). We have indicated the cell type of DMS descending neurons in the text to clarify this. We have also functionally tested DNg27 (instead of DMS descending neurons suggested by the reviewer) using optogenetic and chemogenetic approaches for effects in feeding, food preference, starvation survival, egg laying and flight (new Figure 8 and Figure 8 Supplement 1). While we expected DNg27 to influence flight based on our connectome analyses, we do not see any phenotype in our free flight setup following DNg27 activation (new Figure 8 Supplement 1). However, this could be due to the split GAL4 driver used being very weak (new Figure 8 Supplement 2). This is also supported by the egg-laying assay where DNg27 inactivation only produces a phenotype after day 8 (Figure 8). Since we currently don’t have access to another driver to specifically target DNg27, we are unable to validate our results in the free flight setup using an independent driver.

(3) The authors describe a sparse olfactory input pathway to NSCs, with emphasis on odours playing major roles. However, the physiological consequences of these connections are not explored in detail. Authors should use ORN/AL stimulation (e.g., using optogenetics) to explore how odour sensory pathways affect hormonal secretion in NSCs.

We acknowledge the reviewer’s interest in the physiological consequences of the olfactory to NSC pathways identified in our study. While we agree that exploring how specific odors modulate neuroendocrine output is a logical next step, we believe that such experiments are currently unfeasible due to significant technical and biological constraints as highlighted above for taste neurons. Hence, we calculated the influence of olfactory receptor neuron activation on different NSC classes using an approach based on linear dynamical modeling (new Figure 6 and Figure 6 Supplement 1). Our analysis reveals that smell has a weaker influence on NSC compared to taste.

(4) The authors present a large amount of nice yet complex data, which can be difficult to navigate through and is sometimes hard to follow. Consider adding more schematic diagrams to summarize the key pathways and interactions between NSC types and their inputs/outputs.

We thank the reviewer for this suggestion. We have now included a figure (new Figure 10) which summarizes the main findings from our manuscript and places them within the larger context of neuroendocrine signaling in adult *Drosophila* in relation to other studies.
